# Repair of Bulk-Fill and Nanohybrid Resin Composites: Effect of Surface Conditioning, Adhesive Promoters, and Long-Term Aging

**DOI:** 10.3390/ma15134688

**Published:** 2022-07-04

**Authors:** Muhittin Ugurlu, Nadin Al-Haj Husain, Mutlu Özcan

**Affiliations:** 1 Department of Restorative Dentistry, Faculty of Dentistry, Süleyman Demirel University, Isparta 32200, Turkey; 2 Department of Reconstructive Dentistry and Gerodontology, School of Dental Medicine, University of Bern, 3010 Bern, Switzerland; nadin.al-haj-husain@zmk.unibe.ch; 3 Division of Dental Biomaterials, Clinic of Reconstructive Dentistry, Center of Dental Medicine, University of Zurich, 8032 Zurich, Switzerland; mutlu.ozcan@zzm.uzh.ch

**Keywords:** air-abrasion, aged resin composite, dental materials, diamond bur, durability, minimally invasive dentistry, restorative dentistry, silane, universal adhesive

## Abstract

The aim of the study was to investigate the effect of different repair procedures on the repair bond strength of bulk-fill and nanohybrid resin composites after different aging periods. The resin composite blocks (8 × 8 × 4 mm^3^) were prepared from a bulk-fill (reliaFIL Bulk) and a nanohybrid (reliaFIL LC) resin composite and grouped according to aging duration (6 months, 1 year, and 2 years). Following aging, the blocks were assigned to different surface treatments; air-abrasion with aluminum oxide powder, roughening with a diamond bur, and no treatment. After cleansing with phosphoric acid, a silane layer (Porcelain Primer) was applied on the surface of half of the specimens in each group. The specimens were subdivided into two groups (n = 5): Scotchbond Universal (3M Oral Care) and All-Bond Universal (Bisco). The blocks were repaired with the nanohybrid composite (8 × 8 × 8 mm ^3^). The repaired specimens were stored in distilled water (37 °C/24 h) and segmented into beams. Half of the beams were immediately subjected to microtensile μTBS testing (1 mm/min), while the other half was stored in distilled water (37 °C) for 6 months before testing. Failure modes were analyzed using stereomicroscope and SEM. Statistical analyses were performed with ANOVA and least significant difference tests (LSD) tests (*p* = 0.05). The extension of aging periods (6 months, 1 year, and 2 years) reduced the repair bond strength in some groups for both resin composites (*p* < 0.05). The air-abrasion and bur roughening improved the repair bond strength (*p* < 0.05). The silane application did not influence the repair bond strength and durability (*p* > 0.05). There was no difference among the universal adhesives in the same surface treatment groups (*p* > 0.05). The mechanical roughening treatments are necessary for the repair of resin composite. The universal adhesives might be used for the repair of resin composites regardless of silane content without prior silane application.

## 1. Introduction

The resin composites are frequently used as universal restorative materials for both the restoration of anterior and posterior teeth due to their superior mechanical and esthetic properties [1,2]. There is a high diversity of resin composites available in the market. The nanohybrid composites have commonly been employed because of low polymerization shrinkage and high polishability [3]. The use of bulk-fill resin composites has also been increasing due to the ease of their application [4,5]. However, the aged composite restorations might fail because of different reasons, such as secondary caries, wear, discoloration, chipping, and bulk fracture [1,6,7]. The repair of failed composite restorations is recommended because it is a more conservative, cost-effective, and time-saving treatment approach [7,8,9,10,11]. It has also been reported that the repair improved the clinical longevity of composite restorations [12,13]. 

Several methods have previously been developed and tested to obtain higher repair bond strength of resin composites [7,8]. Previous studies have usually evaluated the physical and chemical treatments performed on the aged resin composites to improve the repair bond strength [8,14]. The physical treatments increase the mechanical interlocking between the resin composites by roughening the surface of the aged composite and enhancing the bonding area, whereas the chemical agents provide a chemical coupling at the interface among the materials [7,8]. It has been stated that the surface roughening treatments are necessary for composite repair procedures [3,15,16]. The most widely used techniques to treat the old composite surface are alumina oxide sandblasting and roughening with bur before chemical treatment with a silane coupling agent or an adhesive [6,15,17,18,19,20,21,22]. 

The silane application before an adhesive might increase the repair bond strength of resin composite [18,22,23,24]. The application of silane on the repair surface may enhance the wetting ability of the aged composite surface and promote chemical bonding between exposed silicate-containing filler particles of the aged composite surface and the resin matrix of the fresh resin layer by forming siloxane bonds [3,7,8,14]. The existence of an adhesive layer additionally has a major role in enhancing the repair potential of aged composites [2,7,16,25]. The use of universal adhesives has become widespread nowadays [26]. The universal adhesives might achieve reliable and stable bond strength for various materials, such as metals, zirconia, porcelain, and composite in addition to enamel and dentin [26]. A silane coupling agent has been added to the composition of some universal adhesives to improve the adhesion of different materials without requiring a separate silane application [23,26,27]. It has been reported that a universal adhesive containing silane provided higher repair bond strength of the resin composites than a silane-free universal adhesive regardless of the silane application beforehand [27]. Nevertheless, it has also been concluded that the silane content of universal adhesive did not affect the composite repair bond strength [5]. There is still not any gold standard procedure for physical and chemical treating of the aged composite surfaces before repair, although various repair strategies are available.

The aging of composites before the repair procedures is crucial to determine the clinical repair potential of a resin composite, because the aging significantly influences the longevity of composite restorations [3,7]. The bond strength of fresh composite compared to the aged composite considerably drops [6,10]. Water aging and water uptake influence the polymer structure of resin composites, thus affecting repair potential [10,11]. During aging, the resin matrix absorbs water, so the unreacted monomers that are important for repair performance leach from the material [2,10]. The effect of short-term water aging on the repair potential of resin composite has been evaluated [2,7,10], but the failures of composite restorations which need to be repaired occur in medium- or long-term clinical situations [3,7,8]. Nonetheless, it is not clear which repair technique is more effective after different long-term aging periods.

Therefore, this study aimed to evaluate the effect of different repair procedures on the repair bond strength of bulk-fill and nanohybrid resin composites after different aging periods. The null hypotheses of this study were (1) that there would not be a difference between the repair bond strength of bulk-fill and nanohybrid resin composites after different aging periods, (2) the prolongation of aging periods would not influence the repair bond strength of bulk-fill and nanohybrid resin composites, and (3) that the different repair procedures would not influence the repair bond strength of bulk-fill and nanohybrid resin composites after different aging periods.

## 2. Material and Methods

### 2.1. Specimen Preparation

**Figure 1:**
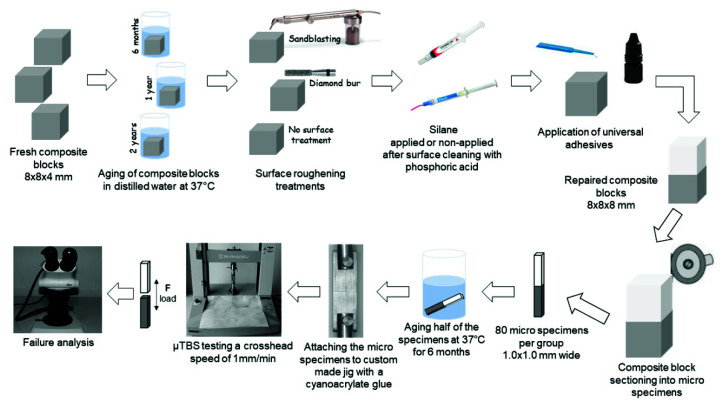
Schematic illustrating the experimental study design.

The materials used in this study, their composition, and application procedures are listed in Table 1. The schematic diagram of the study protocol is shown in Figure 1. A total of 360 resin composite blocks (8 × 8 × 4 mm^3^) were prepared from a bulk-fill (reliaFIL Bulk; Advanced Healthcare Ltd., Tonbridge, UK, Universal shade) and a nanohybrid resin composite (reliaFIL LC; Advanced Healthcare Ltd., Tonbridge, UK, A3) using silicone molds (n_bulk-fill_ = 180, n_nanohybrid_ = 180). The bulk-fill composite was placed 4-mm thick into the mold, and the nanohybrid composite was injected in two layers of 2 mm according to the manufacturer’s instructions. A Mylar strip (SS White Co., Philadelphia, PA, USA) was compressed on top of the mold surface with glass plates to create a flat superficial layer. The resin composites were polymerized through the glass plate using a LED light-curing unit (Smartlite Focus; Dentsply, Milford, DE, USA; light intensity 1000 mW/cm^2^) according to the manufacturer’s instructions. Each composite block was removed from the mold and the surfaces of all the specimens were polished with a multi-step polishing system (Super Snap Rainbow Technique Kit, Shofu, Kyoto, Japan, Lot: 0413007). After each polishing step, all the specimens were thoroughly rinsed with water and air-dried to remove debris. The composite blocks were randomly assigned into aging periods of 6 months, 1 year, and 2 years.

The prepared composite blocks were randomly divided into three aging periods. The composite blocks were stored in distilled water at 37 °C, for the 6-month, 1-year, and 2-year aging periods. The distilled water was changed weekly.

### 2.2. Surface Treatments and Repair Procedure

After the storage periods, the composite blocks were divided into three surface treatment groups: sandblasting with 50 µm aluminum oxide (Al2O3) powder for 10 s at a working distance of 5 mm at a pressure of 5.5 Pascal (Pa) with an intraoral sandblaster (Microetcher II, Danville Engineering Inc., San Ramon, CA, USA); roughening the surface with a fine-grit diamond bur (Komet, Lemgo, Germany) for 10 s under water cooling; and no surface treatment. A 35% phosphoric acid etchant (Scotchbond Etchant Gel; 3M ESPE St Paul, MN, USA) was applied for 30 s to clean the surface of all specimens. After water-rinsing and air-drying, a pre-hydrolyzed silane solution (Porcelain Primer; Bisco, Schaumburg, IL, USA) was applied on the surface of half of the specimens in each group according to the manufacturer′s instructions. The specimens were randomly subdivided into two groups considering different adhesive systems (n = 5): Scotchbond Universal (3M Oral Care, St. Paul, MN, USA) and All-Bond Universal (Bisco, Schaumburg, IL, USA). The adhesives were employed based on the manufacturer’s instructions. After surface treatment and adhesive application, a silicon mold of 8 × 8 × 8 mm^3^ was used to standardize the insertion of 4 mm of fresh resin composite to the aged composite block. Each specimen was repaired with the nanohybrid composite of easily distinguished shade (A1). The composite was inserted in two horizontal layers and light-cured for 20 s per layer. After removing the mold, the specimens were light-cured for 20 s from all four lateral sides. The repaired composite blocks were stored in distilled water for 24 h at 37 °C. After storage, the composite blocks were fixated with a cyanoacrylate glue (Loctite Super Glue, Henkel, Germany) to over a metallic base that was attached to a sectioning machine (Minitom, Struers, Denmark). The blocks were positioned as perpendicular to the diamond disc of the machine. The first section, measuring approximately 1 mm, was discarded. A total of 16 beams with a cross-sectional area of approximately 1 mm^2^ were produced from each block. Half of the beams acquired from each block were used to measure the immediate μTBS; the other half were stored in distilled water for 6 months at 37 °C and tested with the same protocol to determine the aged μTBS. Forty μTBS beams were tested per each experimental group (n = 40).

### 2.3. Microtensile Bond Strength Test

After the exact dimension of each beam was recorded with the digital caliper, they were attached to a custom-made microtensile testing jig with cyanoacrylate glue (Loctite Super Glue, Henkel, Germany) and stressed at a crosshead speed of 1 mm/min until failure in a universal testing machine (Autograph AGS-X; Shimadzu, Kyoto, Japan). The mean μTBS was calculated in MPa, as derived from dividing the imposed force (in N) at the time of fracture by the bond area (in mm^2^). When specimens failed before actual testing (pre-test failures, ptf), they were included as 0 MPa in the calculation of the mean μTBS.

### 2.4. Failure Analyses 

The failure modes were analyzed under 80× magnification using a stereomicroscope. The failure mode was categorized as adhesive failure (interfacial failure), cohesive failure in original composite, cohesive failure in repair composite, and mixed failure (partially adhesive and partially cohesive failure). A few representative samples were chosen for scanning electron microscopy analysis. The specimens were placed in an aluminum sample holder and fixed with carbon tape and viewed with a scanning electron microscope (SEM, Quanta Feg 250, FEI, Eindhoven, The Netherlands).

### 2.5. Micromorphological Analysis 

Three 5 × 2 mm disk-shaped composite disks were prepared from each resin composite using Teflon molds, Mylar strip, and glass plates to analyze the surface after roughening treatments and viewed under SEM.

### 2.6. Statistical Analyses 

The mean of μTBS of the beams producing from the same composite block was calculated, and this mean bond strength was taken as one unit for statistical analysis. Statistical analyses were made with the SPSS Program, version 20.0 (Statistical Package for the Social Sciences; SPSS, Chicago, IL, USA). The Kolmogorov–Smirnov and Levene’s test were used to test the normality of data distribution and homogeneity of variances, respectively. Data were analyzed with repeated measures ANOVA, considering the composite type, aging time of the composite, surface treatments, silane application, adhesives, and storage time as independent factors and repair bond strength as the dependent variable. The LSD test was used for post-hoc comparisons. The *p*-value less than 0.05 was considered statistically significant for all statistical analyses.

## 3. Results

The ANOVA revealed statistically significant differences for aging time (*p* = 0.000), surface treatment (*p* = 0.000), silane application (*p* = 0.000), adhesive (*p* = 0.000), and storage time (0.000), but not for composite type (*p* = 0.580). Moreover, no significant interaction was found between the factors (Table 2).

The overall mean μTBS of all experimental groups and standard deviations, including the results of multiple comparisons statistical analysis, are detailed in Table 3 for bulk-fill resin composite and Table 4 for nanohybrid resin composite.

During all test periods, a significant difference between the repair bond strength of bulk-fill and nanohybrid resin composite was not observed (*p* > 0.05). The groups without any surface treatment showed the lowest repair bond strength values for both resin composites (*p* < 0.05). The air-abraded and bur roughening groups attained similar repair bond strength values for both resin composites (*p* > 0.05). The prolongation of aging periods from 6 months to 1 and 2 years caused a decrease in the repair bond strength in some groups for both resin composites (*p* < 0.05). The application of silane before adhesives did not influence the repair bond strength and durability for both resin composites (*p* > 0.05). There was no statistically significant difference between the adhesives in the same surface treatment groups (*p* > 0.05). The immediate (24 h) and aged (6 months) repair bond strengths were not different in all experimental groups except some groups without surface treatment (*p* > 0.05).

The distribution of failure modes is revealed in Table 5.

**Figure 2:**
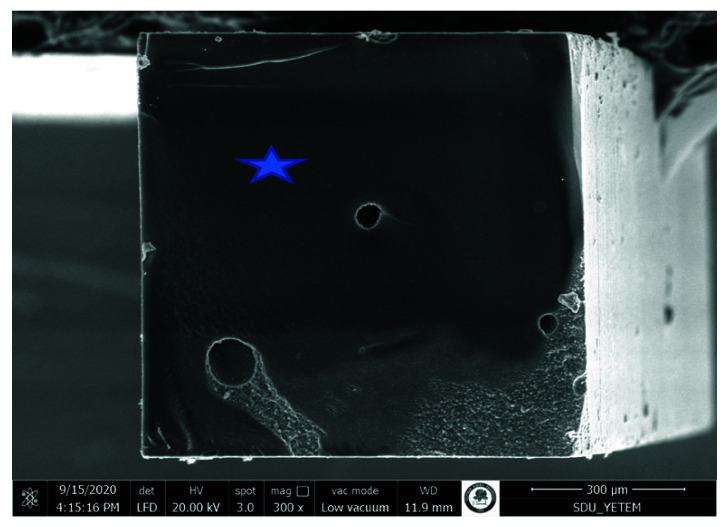
SEM photomicrograph of the fracture surface (aged composite side). The specimen was obtained from 6 months aging of nanohybrid composite, sandblasting, silane applied, Scotchbond Universal group. An adhesive failure pattern was revealed. AL: Adhesive layer on the aged composite surface. RC: Fresh resin composite on the aged composite surface. Note the smooth surface, which did not contribute to micro-mechanical retention (*).

**Figure 3:**
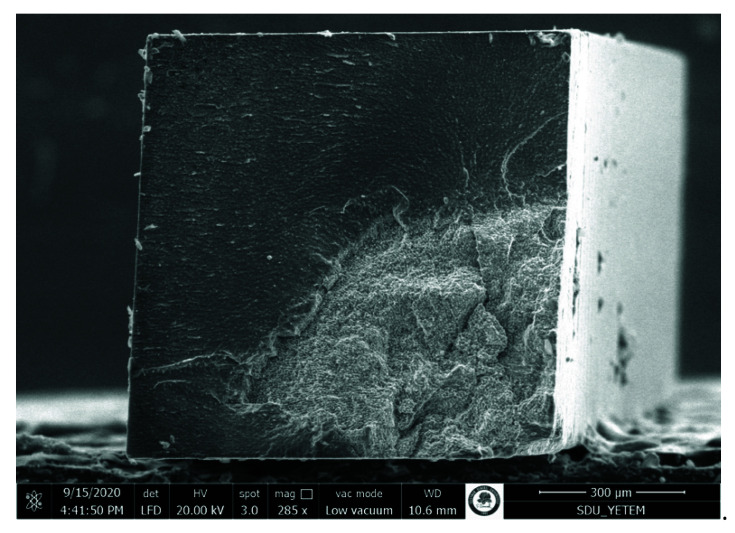
SEM photomicrograph of the fracture surface (aged composite side). The specimen was obtained from 6-month aging of nanohybrid composite, sandblasting, silane applied, All Bond Universal group. A mixed failure pattern was revealed. AL: Adhesive layer on the aged composite surface. RC: Fresh resin composite on the aged composite surface.

**Figure 4:**
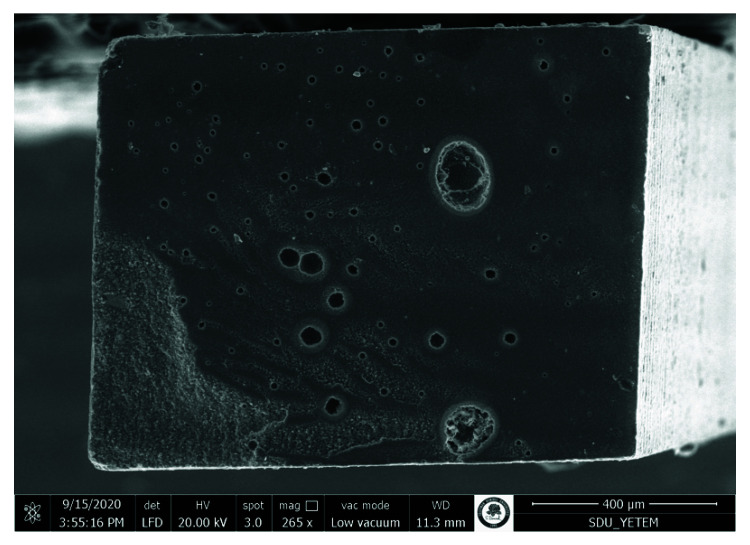
SEM photomicrograph of the fracture surface (aged composite side). The specimen was obtained from 1-year aging of bulk-fill resin composite, sandblasting, silane applied, Scotchbond Universal group. An adhesive failure pattern was revealed.

**Figure 5:**
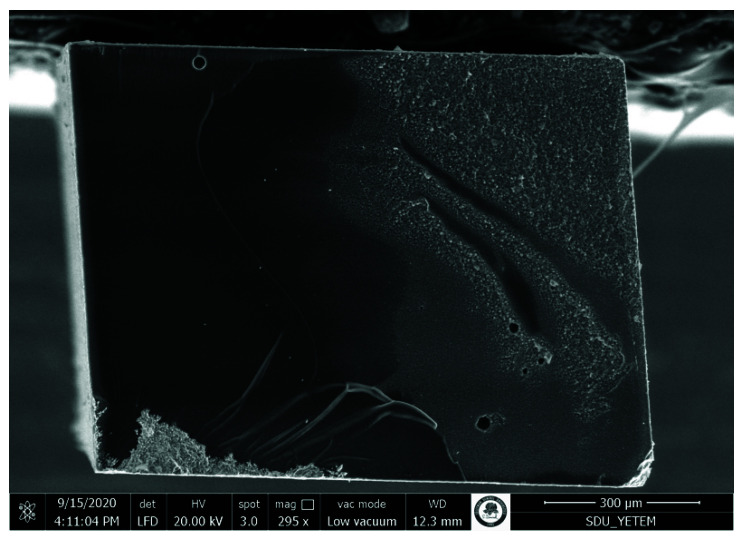
SEM photomicrograph of the fracture surface (aged composite side). The specimen was obtained from 2-year aging of bulk-fill resin composite, sandblasting, silane applied, All Bond Universal group. A mixed failure pattern was revealed.

Pretest failures were detected within only the no surface treatment groups. The adhesive failures were generally more common. The rate of adhesive failure mode was higher in the no surface treatment groups. The cohesive failures were observed more in the aged original composite. The number of cohesive failures in the aged original composite enhanced with the prolongation of aging periods. The representative SEM photomicrographs are presented in Figure 2, Figure 3, Figure 4 and Figure 5. 

## 4. Discussion

In dentistry, several types of resin composites are used for restoration of teeth. The conventional resin composites are applied with the incremental placement technique to reduce polymerization shrinkage stress [28]. This technique may be time-consuming, especially for deep cavities in the posterior area [28]. The bulk-fill resin composites which have greater depth of cure and may be placed in one increment of 4 mm have been developed to overcome this problem [4]. The bulk-fill resin composites have similar chemical compositions with conventional resin composites excluding some modifications in photo initiators and fillers [4]. The composite restorations may fail regardless of the composite type [1,29]. Failed composite restorations may be repaired with various treatment approaches [7,8]. In the present study, the effect of different repair procedures on the repair bond strength of bulk-fill and nanohybrid resin composites after different aging periods was evaluated. The repair bond strengths of bulk-fill and nanohybrid resin composites were not different. Therefore, the first null hypothesis that there would not be a difference between the repair bond strength of bulk-fill and nanohybrid resin composites after different aging periods was accepted. The success of composite repair depends on the chemical composition of the resin composite [10]. The chemical content of the employed resin composites in this study is similar. It has been stated that the homogeneity of aged and freshly composite could be beneficial to improve the copolymerization performance between their resin matrix monomers [10]. Nonetheless, it has been reported the bulk-fill composite might be repaired with conventional resin composites [5,29,30], which could be approved by this study, when bulk-fill and nanohybrid resin composites were used.

Aging the resin composites is needed for assessment of repair bond strength of them to mimic the aging of composite restorations in the oral environment [10,31]. However, there is not any gold standard procedure for the aging of resin composites to simulate oral conditions, although different in vitro aging methods are available [5,29]. Water aging is one of the in vitro aging methods [2,10]. The unreacted monomers in the matrix of resin composite have a crucial role in repair bonding performance in early periods [10,11], as during aging, they ooze from the resin composite, and the resin matrix absorbs water [11,32]. The absorbed water might cause a reduction in the wettability of freshly resin composite, which is used as a repair material, therefore decreasing the repair bond strength [10,32]. The prolongation of aging time might induce an increase in water sorption [33], thus affecting the repair bond strength of resin composite [10]. It has been stated that aging for 1 year might simulate the composite degradation that occurs in the oral environment [32]. In the present study, the repair bond strength of bulk-fill and nanohybrid resin composites was evaluated after 6 months (6M), 1 (1Y), and 2 years (2Y). The extension of aging period from 6 months to 1 and 2 years decreased the repair bond strength of the resin composites in some experimental groups significantly (bulk-fill resin composite: 1—sandblasting: ABU + silane, SBU/ABU—silane, immediate 6M -> 1Y; 2—bur: ABU + silane immediate 1Y -> 2Y and SBU/ABU—silane, immediate 6M -> 1Y; 3—no treatment, SBU + silane, immediate 1Y -> 2Y; aged 6M -> 1Y and ABU + silane, immediate 6M -> 2Y, SBU—silane, immediate 6M -> 1Y; aged 6M -> 1Y; aged 1Y -> 2Y and ABU—silane, aged 1Y -> 2Y; (Nanohybrid resin composite: 1—sandblasting: ABU—silane, immediate 6M -> 1Y; 2—bur: ABU—silane, immediate 6M -> 1Y; 3—no treatment, SBU/ABU—silane, aged 6M -> 1Y).

Therefore, the second null hypothesis that the prolongation of aging periods would not influence the repair bond strength of bulk-fill and nanohybrid resin composites was partially rejected. The decrease might result from increased water absorption with the prolongation of the aging period. Unfortunately, the water sorption was not evaluated in the present study. It has previously been reported that the extension of water storage periods reduced the repair bond strength [10,32]. Moreover, it has been stated that the repair of restoration might not be a successful treatment option when the composite restoration has exposure to the oral environment for a long period [31].

During repair, providing a mechanical interlocking via roughening treatments is the most crucial factor for obtaining a reliable bonding between an aged and a fresh composite [6,16]. Previous studies have tested various roughening procedures, including sandblasting with aluminum oxide particles, roughening with diamond burs, lasers, and etching with hydrofluoric acid [23,34,35]. However, air-abrasion with aluminum oxide particles and roughening with diamond burs have usually been preferred [6,19,20,22]. In this study, the lowest repair bond strength values were found in the groups without any surface treatment at all test periods. This result is in agreement with previous studies [6,20,21]. This is due to the poor micro-mechanical adhesion as observed on the smooth surface in SEM image in Figure 2. The micro-mechanical interlocking is the main bonding mechanism underlying composite repair [16,22]. The sandblasting with aluminum oxide particles and roughening with diamond bur might induce an increase in the surface roughness of the composite surface, thus improving repair bond strength by promoting micro-mechanical interlocking, as reported previously [6,22]. Adequate micro-mechanical retention might not occur when no mechanical roughening is performed on the composite surface. The repair bond strength values obtained from sandblasting with aluminum oxide particles and roughening with diamond bur were similar at all test periods. Previous studies have also concluded that there was no difference in the repair bond strength values provided by the alumina sandblasting and bur roughening [6,22].

The beneficial effects of silane application at the composite repair have previously been reported [5,18,22,24]. Silane might improve the wetting ability of the adhesives to a roughened composite surface [23,24]. Besides, it may also provide a chemical bonding between the filler particles of aged composite and resin matrix of fresh composite [18,23]. However, it has been concluded that the silane application reduced the repair bond strength [19] and did not influence it [16,34,36,37]. In this study, the silane application did not affect the repair bond strength and durability. The differences in the results of studies may be due to differences in methods and used materials. It has been stated that the chemical content of the silane and the filler type of the resin composite may change the effectiveness of the silane on the composite repair [19,23].

For a successful repair of resin composites, the use of adhesives is required after mechanical pretreatments [37,38]. The adhesives promote the penetration capacity of freshly resin composite into the surface microstructure of the aged composite due to its high viscosity [38]. Additionally, the use of adhesives enhances the chemical bonding potential of the aged composite by providing a chemical interaction between the exposed fillers of aged composites and the resin matrix of freshly composite [25,35]. In this study, two universal adhesives were employed and, furthermore, no difference was determined in the repair bond strength values obtained by the adhesives. Scotchbond Universal and All-Bond Universal have 10-MDP in their chemical content as a functional monomer. This functional monomer has superior bonding efficacy and it creates a resistant adhesive interface to biodegradation [26]. The phosphate esters of the monomer can directly bond to various materials, such as ceramic, zirconia, and composite [23]. The 10-MDP might bond to aged resin composite because it is a solvating monomer that may penetrate to a cross-linked network [39]. Thereby, it improves the repair bond strength by providing additional chemical bonding [3,39]. Scotchbond Universal furthermore has an organosilane agent in its composition. However, the silane content of universal adhesives might not impact the performance of adhesive for repair bond strength of resin composites [5,37]. The inefficacy of the silane in the content of universal adhesives may result from the low stability of the silane in an acidic adhesive solution [40]. The silanol groups of the silane may undergo dehydroxylation in the acidic adhesive solution containing water; therefore, the bonding capacity may decrease [40]. Furthermore, it has been concluded that the repair bond strength acquired with universal adhesives was not influenced by silane application beforehand [40].

According to the results of this study, the third null hypothesis that the different repair procedures would not influence the repair bond strength of bulk-fill and nanohybrid resin composites after different aging periods was also partially rejected. The ultimate goal of repair of resin composite restorations is to achieve superior bond strength as well as durable bond strength [38]. In the present study, the durable bond strength values were acquired from all the experimental groups, except for some groups without any surface treatments, in agreement with previous studies [21,22,32]. In the present study, the adhesive failure mode was mostly observed failure mode. The prolongation of the aging periods of composites from 6 months to 1 and 2 years increased the cohesive failure mode in the aged composite. It may result from the decreasing cohesive strength of the resin materials by increasing water absorption with the extension of aging time. The absorbed water plasticizes the polymer matrix and diffuses into the silane-treated filler-matrix interface, in doing so inducing hydrolysis within the resin matrix and at the resin-filler interface [25]. The percentage of adhesive failure mode was higher in the groups without any surface treatments. This may be related to a weaker repair interface considering the bond strength values. Notwithstanding, these results were revealed in laboratory conditions, hence further in vitro and clinical studies are required to validate the results.

## 5. Conclusions

The clinical relevance of this study is that bulk-fill resin composites can be repaired with nanohybrid resin composites. The repair of composite restorations presents a feasible and effective treatment option when performed after a shorter period. The mechanical roughening of the aged resin composite is needed to obtain optimal repair bond strength and durability. The universal adhesives can be used for the repair of resin composites regardless of their silane content. Application of silane before universal adhesives did not improve repair bond strength and durability.

## Figures and Tables

**Table 1:** The materials, chemical composition, and application procedure.

Material	Composition	Application Procedure
reliaFIL LC (Advanced Healthcare Ltd., Tonbridge, UK) Batch no:20200228	Bis-GMA, TEGDMA, fluoroboroaluminosilicate glass fillers, photoinitiators	1. Apply the material in thin layers (max. 2 mm) 2. Polymerize the material using a light-curing unit with light output of 1550–550 mW/cm²
reliaFIL Bulk (Advanced Healthcare Ltd., Tonbridge, UK) Batch no:20200728	Bis-GMA, TEGDMA, fluoroaluminosilicate glass fillers, photoinitiators	1. Apply the material in a layer of up to 4 mm depth 2. Polymerize the material using a light-curing unit with light output of 1550 mW/cm²
Porcelain Primer (BISCO, Schaumburg, IL, USA) Lot no:1800003839	3-(Trimethoxysilyl)propyl-2-Methyl-2-Propenoic Acid, ethanol, acetone	1. Apply 1 thin coat to surface and allow to dwell for 30 s 2. Dry with air syringe
Scotchbond Universal (3M Oral Care, St. Paul, MN, USA) Batch no: 602724	10-MDP phosphate monomer, dimethacrylate resins, HEMA, methacrylate-modified polyalkenoic acid copolymer, filler, ethanol, water, initiators, silane	1. Apply the adhesive with a microbrush and rub it in for 20 s 2. Direct a gentle stream of air over the liquid for about 5 s until it no longer moves and the solvent is evaporated completely 3. Light-cure for 10 s
All-Bond Universal (BISCO, Schaumburg, IL, USA) Batch no: 1500005353	10-MDP phosphate monomer, Bis-GMA, HEMA, ethanol, water initiators	1. Apply the adhesive as two separate coats in a scrubbing mode with a microbrush for 10–15 s per coat 2. Dry for at least 10 s 3. Light-cure for 10 s

Composition as provided by the manufacturers: Bis-GMA, bisphenol-glycidyl methacrylate; 10-MDP, 10-methacryloyloxydecyl dihydrogen phosphate; HEMA, hydroxyethylmethacrylate; 4 MET, 4-methacryloxyethyl trimellitate; TEGDMA: Triethylenglykol Dimethacrylate.

**Table 2:** The ANOVA results for microtensile bond strength test.

Source	Sum of Squares	df	Mean Square	F	*p*
Composite type	11.633	1	11.633	0.307	0.580
Aging time	6546.618	2	3273.309	86.434	0.000 *
Surface treatment	83,857.974	2	41,928.987	1107.160	0.000 *
Silane application	960.025	1	960.025	25.350	0.000 *
Adhesive	749.674	1	749.674	19.796	0.000 *
Storage time	742.637	1	742.637	59.959	0.000 *
Interaction	1.101	4	0.275	0.006	1.000

* Statistically significant differences (*p* < 0.05).

**Table 3:** The means and standard deviations (μTBS in MPa ± SD) of repair strength of bulk-fill resin composite for all experimental groups.

	Silan	Adhesive			1 Year		2 Years	
			*Immediate*	*Aged*	*Immediate*	*Aged*	*Immediate*	*Aged*
	Yes	SBU	52.05 ± 6.44 *^,^°	51.32 ± 6.50 *	49.63 ± 5.88 *^,^°	49.22 ± 5.37 *	47.14 ± 5.30 *^,^°	46.07 ± 5.52 *
Sandblasting		ABU	50.23 ± 5.70 *^,^°	49.58 ± 5.83 *	47.91 ± 5.67 *^,^°^,^§	47.32 ± 5.92 *	42.26 ± 5.43 *^,^§	41.72 ± 5.30 *
	No	SBU	51.69 ± 6.86*°	50.74 ± 6.52 *	45.23 ± 5.45 *^,^°^,^§	44.83 ± 5.21 *	41.93 ± 5.79 *^,^§	40.37 ± 5.16 *
		ABU	50.18 ± 5.62 *^,^°	49.76 ± 5.52 *	43.69 ± 5.32 *^,^°^,^§	42.36 ± 5.15 *	39.04 ± 4.98 *^,^§	38.14 ± 4.75 *
	Yes	SBU	50.87 ± 5.78 *^,^°	50.28 ± 5.36 *	49.06 ± 5.26 *^,^°	48.27 ± 5.47 *	44.85 ± 5.38 *^,^°	44.50 ± 5.51 *
Bur		ABU	48.51 ± 5.45 *^,^°	48.16 ± 5.31 *	46.33 ± 5.46 *^,^°^,^§	45.82 ± 5.34 *	40.76 ± 5.08 *^,^§	40.58 ± 5.43 *
	No	SBU	50.16 ± 5.62 *^,^°	49.42 ± 5.61 *	43.82 ± 5.66 *^,^§	43.53 ± 5.23 *	41.06 ± 5.17 *^,^§	40.22 ± 5.22 *
		ABU	49.30 ± 5.29 *^,^°	47.80 ± 5.36 *	41.99 ± 5.12 *^,^§	41.64 ± 5.24 *	38.22 ± 5.07 *^,^§	36.93 ± 5.09 *
	Yes	SBU	29.10 ± 4.42 *^,^°	22.94 ± 4.47 #	28.11 ± 4.38 *^,^°	22.78 ± 4.17 *	21.80 ± 4.02 *^,^§	17.06 ± 5.04 *
No treatment		ABU	27.79 ± 4.32 *^,^°	22.63 ± 4.46 *	25.90 ± 4.14 *^,^°^,^§	21.40 ± 4.25 *	21.57 ± 4.47 *^,^§	16.88 ± 4.44 *
	No	SBU	29.33 ± 4.29 *°	22.33 ± 4.42 *	26.00 ± 4.36 *^,^°^,^§	21.08 ± 4.25 #	21.69 ± 4.17 *^,^§	15.86 ± 5.84 *
		ABU	26.07 ± 4.02 *^,^°	22.00 ± 4.25 #	23.31 ± 4.40 *^,^°	19.70 ± 4.06 #	21.32 ± 4.31 *^,^°	16.67 ± 4.83 *

SD, standard deviation; n = 40, total number of specimens for each experimental group; SBU, Scotchbond Universal; ABU, All Bond Universal; same small letter in the columns indicates no statistically significant difference between the immediate (24 h) and aged (6 m) bond strength values of each experimental group; same capital letter in the columns indicates no statistically significant difference in the immediate (24 h) bond strength values acquired after different aging periods of resin composite. The groups without any surface treatment showed the lowest repair bond strength values at all test periods (*p* < 0.05); however, similar repair bond strength values were acquired by the sandblasting and bur roughening (*p* > 0.05). The application of silane before adhesives did not influence the repair bond strength at all test periods (*p* > 0.05). There was no statistically significant difference between the adhesives in the same surface treatment groups at all test periods (*p* > 0.05). The symbols *, °, § and # imply statistical significance.

**Table 4:** The means and standard deviations (μTBS in MPa ± SD) of repair strength of nanohybrid resin composite for all experimental groups.

	Silan	Adhesive			1 Year		2 Years	
			*Immediate*	*Aged*	*Immediate*	*Aged*	*Immediate*	*Aged*
	Yes	SBU	51.47 ± 7.84 *^,^°	50.87 ± 7.13 *	48.95 ± 5.38 *^,^°	48.67 ± 5.31 *	46.03 ± 5.20 *^,^°	45.61 ± 4.80*
Sandblasting		ABU	48.68 ± 6.26 *^,^°	48.31 ± 6.35 *	47.23 ± 5.84 *^,^°	46.76 ± 5.89 *	42.68 ± 5.87 *^,^°	41.66 ± 5.29 *
	No	SBU	50.44 ± 7.69 *^,^°	50.01 ± 7.25 *	44.95 ± 5.40 *^,^°	44.10 ± 5.46 *	41.63 ± 5.88 *^,^°	40.30 ± 5.09 *
		ABU	49.41 ± 5.56 *^,^°	48.87 ± 5.12 *	42.78 ± 5.72 *^,^°§	41.92 ± 5.30 *	38.48 ± 5.55 *^,^§	37.66 ± 5.19 *
	Yes	SBU	50.16 ± 6.70 *^,^°	49.99 ± 5.88 *	48.65 ± 5.61 *^,^°	47.81 ± 5.42 *	45.25 ± 5.31 *^,^°	45.24 ± 5.23 *
Bur		ABU	47.72 ± 6.35 *^,^°	47.44 ± 6.07 *	46.03 ± 6.18 *^,^°	45.76 ± 5.27 *	41.21 ± 5.78 *^,^°	40.91 ± 5.73 *
	No	SBU	49.41 ± 6.34 *^,^°	49.09 ± 6.21 *	44.07 ± 5.95 *^,^°	43.70 ± 5.11 *	41.34 ± 5.48 *^,^°	40.06 ± 5.01 *
		ABU	48.62 ± 5.46 *^,^°	47.27 ± 5.26 *	42.58 ± 5.90 *^,^°^,^§	41.86 ± 5.07 *	37.46 ± 5.01 *^,^§	36.66 ± 5.26 *
	Yes	SBU	28.13 ± 6.01 *^,^°	23.92 ± 5.06 *	27.96 ± 5.74 *^,^°	22.80 ± 4.19 *	22.34 ± 5.00 *^,^°	18.06 ± 5.26 *
No treatment		ABU	26.07 ± 4.36 *^,^°	22.73 ± 4.74 *	25.89 ± 4.13 *^,^°	21.64 ± 4.52 *	21.50 ± 4.74 *^,^°	17.28 ± 4.99 *
	No	SBU	27.97 ± 5.95 *^,^°	22.70 ± 5.06 *	25.78 ± 4.77 *^,^°	21.18 ± 4.39 #	22.23 ± 5.13 *^,^°	16.91 ± 6.37 *
		ABU	25.86 ± 4.40 *^,^°	21.40 ± 4.19 #	23.61 ± 4.63 *^,^°	19.80 ± 4.20 *	21.31 ± 4.91 *^,^°	16.68 ± 4.84 *

SD, standard deviation; n = 40, total number of specimens for each experimental group; SBU, Scotchbond Universal; ABU, All Bond Universal; same small letter in the columns indicates no statistically significant difference between the immediate (24 h) and aged (6 m) bond strength values of each experimental group; same capital letter in the columns indicates no statistically significant difference in the immediate (24 h) bond strength values acquired after different aging periods of resin composite. The groups without any surface treatment showed the lowest repair bond strength values at all test periods (*p* < 0.05); however, similar repair bond strength values were acquired by the sandblasting and bur roughening (*p* > 0.05). The application of silane before adhesives did not influence the repair bond strength at all test periods (*p* > 0.05). There was no statistically significant difference between the adhesives in the same surface treatment groups at all test periods (*p* > 0.05). The symbols *, °, § and # imply statistical significance.

**Table 5:** The distribution of failure modes of the tested beams.

		Silane	Adhesive			1 Year		2 Years	
				*Immediate*	*Aged*	*Immediate*	*Aged*	*Immediate*	*Aged*
**Bulk-fill composite groups**	**Sandblasting**	Yes	SBU	18/7/4/11	22/5/3/10	13/11/6/10	15/10/7/8	12/13/7/8	13/13/7/7
			ABU	20/7/3/10	21/8/3/8	15/10/7/8	18/9/6/7	11/14/5/10	14/14/4/8
		No	SBU	21/7/5/7	21/9/3/7	16/11/5/8	18/12/5/5	13/13/6/8	13/12/6/9
			ABU	22/5/4/9	20/8/4/8	17/9/4/10	19/10/6/5	11/12/8/9	13/13/5/9
	**Bur**	Yes	SBU	21/6/3/10	22/6/3/9	17/11/5/7	19/11/2/8	10/13/7/10	14/12/7/7
			ABU	20/7/4/9	19/10/4/7	18/10/3/9	20/10/4/6	12/14/7/7	13/12/7/8
		No	SBU	19/8/2/11	18/9/2/11	16/10/6/8	20/11/2/7	11/14/7/8	14/14/5/7
			ABU	18/9/3/10	20/7/4/9	18/12/5/5	18/10/5/7	10/13/9/8	13/11/8/8
	**No treatment**	Yes	SBU	32/1/0/4 ptf = 3	34/1/1/2 ptf = 2	30/2/1/5 ptf = 2	34/1/0/2 ptf = 3	33/2/0/2 ptf = 3	35/1/0/0 ptf = 4
			ABU	32/2/1/3 ptf = 2	33/1/1/2 ptf = 3	31/1/1/5 ptf = 2	35/1/0/0 ptf = 4	32/2/0/3 ptf = 3	36/0/0/0 ptf = 4
		No	SBU	29/3/1/4 ptf = 3	32/2/1/2 ptf = 3	30/1/1/6 ptf=2	33/1/0/2 ptf = 4	31/2/0/3 ptf = 4	35/0/0/1 ptf = 4
			ABU	28/1/1/7 ptf = 3	34/1/1/1 ptf = 3	33/1/1/1 ptf = 4	32/2/1/2 ptf = 3	33/1/0/3 ptf = 4	34/0/0/1 ptf = 5
**Nano hybrid composite groups**	**Sandblasting**	Yes	SBU	21/6/4/9	20/7/4/9	14/10/6/10	16/10/5/9	13/11/6/10	15/12/4/9
			ABU	19/8/3/10	19/10/3/8	13/11/6/8	17/9/5/9	13/12/4/11	14/14/4/8
		No	SBU	20/7/3/9	22/8/2/8	16/11/4/7	18/12/3/7	14/14/4/8	16/12/4/8
			ABU	20/6/4/10	21/7/4/8	18/9/3/10	19/10/4/7	13/12/7/8	15/13/5/7
	**Bur**	Yes	SBU	19/8/3/10	20/7/3/10	16/11/4/9	20/10/3/7	13/13/5/9	16/12/4/8
			ABU	22/5/4/9	23/6/4/7	17/10/4/9	20/10/5/5	11/14/7/8	14/12/6/8
		No	SBU	21/6/2/11	22/9/1/8	18/11/3/8	19/11/3/7	14/13/4/9	14/14/5/7
			ABU	20/7/4/9	22/8/3/7	19/12/3/6	21/10/3/6	13/13/6/8	15/11/5/9
	**No treatment**	Yes	SBU	31/2/1/4 ptf = 2	33/2/0/2 ptf = 3	30/3/0/5 ptf = 2	33/2/0/2 ptf = 3	30/3/0/3 ptf = 4	35/0/0/1 ptf = 4
			ABU	30/2/1/4 ptf = 3	34/2/0/0 ptf = 4	31/2/1/4 ptf = 2	34/1/0/2 ptf = 3	33/4/0/1 ptf = 2	34/0/0/2 ptf = 4
		No	SBU	32/1/0/4 ptf = 3	32/2/1/2 ptf = 3	32/2/1/4 ptf = 3	34/1/0/2 ptf = 3	32/4/0/2 ptf = 2	36/1/0/0 ptf = 3
			ABU	32/2/1/3 ptf = 2	33/2/0/3 ptf = 2	33/1/1/2 ptf = 3	33/2/0/2 ptf = 3	31/3/0/2 ptf = 4	35/0/0/1 ptf = 4

Adhesive failure/Cohesive failure in original composite/Cohesive failure in repair composite/Mixed failure; n = 40, total number of specimens for each experimental group; SBU, Scotchbond Universal; ABU, All Bond Universal; ptf, pretest failures.
